# Quantitative analysis of sensor for pressure waveform measurement

**DOI:** 10.1186/1475-925X-9-6

**Published:** 2010-01-21

**Authors:** Shing-Hong Liu, Chu-Chang Tyan

**Affiliations:** 1 Department of Computer Science and Information Engineering, Chaoyang University of Technology, Taichung, Taiwan; 2 Division of Chinese Medicine, Buddhist Tzu Chi General Hospital, Taipei Branch, Taipei, Taiwan; 3 Department of Chinese Medicine, China Medical University, Taichung, Taiwan

## Abstract

**Background:**

Arterial pressure waveforms contain important diagnostic and physiological information since their contour depends on a healthy cardiovascular system [1]. A sensor was placed at the measured artery and some contact pressure was used to measure the pressure waveform. However, where is the location of the sensor just about enough to detect a complete pressure waveform for the diagnosis? How much contact pressure is needed over the pulse point? These two problems still remain unresolved.

**Method:**

In this study, we propose a quantitative analysis to evaluate the pressure waveform for locating the position and applying the appropriate force between the sensor and the radial artery. The two-axis mechanism and the modified sensor have been designed to estimate the radial arterial width and detect the contact pressure. The template matching method was used to analyze the pressure waveform. In the *X-*axis scan, we found that the arterial diameter changed waveform (ADCW) and the pressure waveform would change from small to large and then back to small again when the sensor was moved across the radial artery. In the *Z*-axis scan, we also found that the ADCW and the pressure waveform would change from small to large and then back to small again when the applied contact pressure continuously increased.

**Results:**

In the *X-*axis scan, the template correlation coefficients of the left and right boundaries of the radial arterial width were 0.987 ± 0.016 and 0.978 ± 0.028, respectively. In the *Z*-axis scan, when the excessive contact pressure was more than 100 mm Hg, the template correlation was below 0.983. In applying force, when using the maximum amplitude as the criteria level, the lower contact pressure (*r *= 0.988 ± 0.004) was better than the higher contact pressure (*r *= 0.976 ± 0.012).

**Conclusions:**

Although, the optimal detective position has to be close to the middle of the radial arterial, the pressure waveform also has a good completeness with a template correlation coefficient of above 0.99 when the position was within ± 1 mm of the middle of the radial arterial range. In applying force, using the maximum amplitude as the criteria level, the lower contact pressure was better than the higher contact pressure.

## 1. Background

Arterial pressure waveforms contain important diagnostic and physiological information since their contour depends on a healthy cardiovascular system [1]. Kelly and Fitchett formulated a mathematical equation for converting radial artery pressure waveforms into central aortic pressure waveforms, and accordingly calculated the central aortic pressure [2]. The pressure waveform can also be used to calculate the augmentation index to estimate the arterial stiffness [3-5]. Cohn *et*. *al*. used pulse wave analysis in screening subjects for early evidence of vascular disease and in monitoring the response to therapy [6]. In 1993, Chen *et al*. used the Fourier transform to study the radial artery waveform, which would be affected by mechanical resonance between the heart and other organs [7]. McLaughlin *et al*. used the piezoelectric sensor to determine the arterial pulse wave velocity [8]. The non-invasive continuous blood pressure monitor uses the artery tonometry to detect the pressure waveform at the radial artery [9,10].

In these studies, a single or a sensor array was placed at the measured artery and some contact pressure was used to detect the pressure waveform. In general, the sensor has to be placed close to the middle of the artery. Under this condition, the sensor can register the maximum amplitude of the pressure waveform. Therefore, authors of the previous studies have always described the optimal measuring position for the sensor as over the artery with light adjustments. Concerning the applied force of the sensor, because the compliance of the artery belongs to a nonlinear curve, the contact pressure will affect the shape of the pressure waveform. Yoon *et. al*. had found that the amplitude of the pressure waveform first increases, reaching a maximum, and then decreases when the contact pressure is continuously increased [11]. Driscoll *et. al*. used the harmonic coefficients to show that pressure pulse contour, pulse wave velocity and harmonic transmission ratios were relatively stable in the brachial and radial arteries of normal subjects over a large range of the force applied at these arteries [12,13]. They also found that the contact pressure at the level of the arterial wall would be larger for subjects with greater amount of subcutaneous tissue and fat over the artery. Moreover, the excessive contact pressure may produce alterations in arterial geometry that can distort the arterial pressure waveform and consequently diminish its value as a diagnostic aid. Liu *et. al*. proposed a two-axis mechanism who used this mechanism to measure the pressure waveform for estimating the myocardial ischemia symptoms [14,15], and to measure the arterial diameter change waveform for estimating the compliance of the radial artery [16]. In these studies, the pressure waveforms were measured when the sensor was placed at the middle of radial artery, and applied an unloading contact pressure.

**Figure 1:**
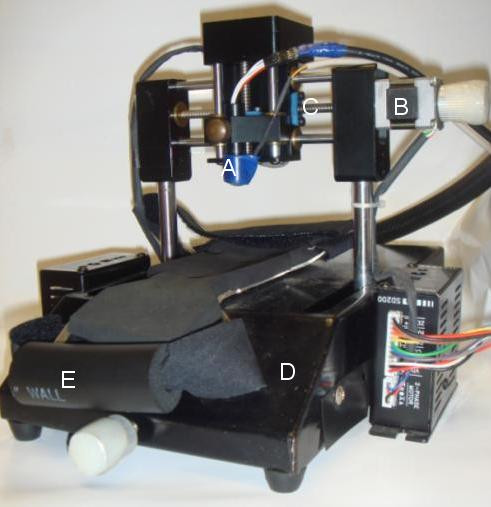
**Schematic of the two-axis mechanism**. A: Sensor; B: Stepping motor: C: *X*-axis screw; D: Platform; E: Handgrip.

However, the question is whether the sensor has to be placed at the middle of artery and the applied force also has to be in an unloading condition for the pressure waveform measurement or not. If the sensor couldn't be placed at middle of artery, where is the location of the sensor just about enough to detect a complete pressure waveform for the diagnosis? Moreover, if the unloading condition couldn't be satisfied, how much would contact pressure be fine? Therefore, in this paper, we studied the distortion of the pressure waveform affected by the sensor's location and contact pressure. We used our designed two-axis mechanism (Pattern Number: I309976, R.O.C.) to quantitatively measure the radial pressure waveform which real photo is shown in Fig 1. A modified sensor that can detect arterial diameter change waveform (ADCW) and pressure waveform with the strain gauge and the piezoresistor, simultaneously was used. In the *X*-axis scanning procedure, we used the template matching method to analyze the distortion degree of the pressure waveform when the sensor was moved across the radial artery. In the *Z*-axis scanning procedure, we also used the template matching method to analyze the distortion degree of the pressure waveform when the contact pressure was continuously increased. This study used 28 patients with untreated, mild or moderate hypertension and 14 normotensive patients to investigate the change in pressure waveform.

## 2. Method

The modified sensor combined a piezoresistor(diameter: 12 mm, range 15 psi; NPI-12, Lucas, USA) with a strain gauge (size: 5 × 2 mm, resistance: 119.8 ± 0.2% Ω, mean ± SD, gage factor: 2.12; KFG-5-120-C1, Kyowa, Japan). Its active surface was semispherical and constructed from soft silicone rubber. We have shown that the sensor could measure the ADCW and pressure waveform simultaneously [14]. The waveform signals were recorded using a digital-to-analog acquisition card (PCI 6014, National Instruments), with LabView software used for data retrieval and manipulation. The analog bandwidth, pressure-sensor gain, strain-gauge gain and sampling frequency were 0.1-40 Hz, 100, 5000 and 500 Hz, respectively. The pressure sensor was calibrated using a mercury-column pressure gauge.

Because the template matching method could compare the correlation between the reference pattern and the test pattern, we used it to analysis the distortion of the pressure waveform affected by the sensor's location and the applied force in this paper. In the registered pressure signal from *X*-axis or *Z*-axis scans, one cycle pressure waveform was chosen as the reference pattern to compare over the pressure waveform. The reference pattern was considered as an optimal waveform in the whole pressure signal. The correlation signal was generated by shifting the reference pattern, *x*, the whole pressure signal, *y*, and simultaneously computing the correlation coefficient *r_xy_*[*k*] for each time index *k *according to, the size of *x *is *N *[17],(1)(2)

### 2.1 Data Collection

The noninvasive pressure waveform usually was used to extract the augmentation index or the pulse waveform velocity [5,8]. These parameters have widely been applied to diagnosis the arterial stiffness in the clinic. In order to increase the experimental reliability, we selected two groups, hypertension and no hypertension, to quantitatively measure pressure waveform. The hypertension group represents whose arteries belong to the high risk of the stiffness. Oppositely, no hypertension group represents whose arteries belong to the normal condition. The experimental protocol was applied to 42 patients (29 men and 13 women) aged 51 ± 11 years (mean ± standard deviation (SD); range: 25 to 85 years) who were selected by the doctor according to their anamneses. All patients underwent blood pressure tests. The blood pressure was measured with an electric sphygmomanometer (DINAMAP PROCARE 100, GE Medical Systems, U.S.A.) in the left arm of each patient. Hypertension was defined as systolic blood pressure (SBP) when it exceeded 140 mm Hg and/or diastolic bloody pressure (DBP) when it exceeded 90 mm Hg. The 14 patients (10 men and 4 women) are no hypertension. The hypertensive group has 28 patients (19 men and 9 women) whose blood pressures are always in the mild or moderate range. The patients' characteristics are shown in Table 1. All patients were asked to stop smoking and alcohol intake, and avoid consuming stimulants (e.g., coffee and tea) and medications for at least 24 hours before participating in the experiments. They rested in a temperature-controlled room (at 22 ± 1°C) for 30 minutes before the waveform examination was performed. During the waveform measurement, each patient was asked to sit on an adjustable-height chair. The patient's right hand was placed on a table at the same horizontal height as the heart with the palm pointing upwards and the wrist resting on a soft pillow. The arm was positioned between the platform and the sensor and was held in place with a Velcro strap. The hand grasped the handgrip to let the radial artery appear. The radial styloid process was used as the reference point to detect the pressure waveform. Measurement began from the *X*-axis - the entire procedure is explained in Appendix A.

**Table 1:** Characteristics of subjects

	Total (*n *= 42)	Hypertension (*n *= 28)
Sex (men/women)	29/13	19/9
Age (Years)	51 ± 11	52 ± 13
BMI (kg/m^2^)	27 ± 3	27 ± 3
SBP (mm Hg)	140 ± 21	151 ± 16
DBP (mm Hg)	84 ± 16	89 ± 15
MAP (mm Hg)	103 ± 16	110 ± 14

BMI, body mass index; SBP, systolic blood pressure; DBP, diastolic blood pressure; MAP, mean arterial pressure.

### 2.2 Pressure Analysis in *X*-axis

In the *X*-axis scan, each step of the stepping motor corresponded to a movement of 0.25 mm, and the recording time was 4 seconds. There were three to four complete cycles for the beat pulses. The scanning direction was from left to right. The contact pressure was kept within a stable range for each segment. According the vascular geometry analysis, the ADCW would become large and then small (see Appendix B). A Gaussian equation could describe the envelope of the ADCW [14],(3)

where *ADCW*_*max *_is the maximum value, *X *is the location of the sensor, *X*_0 _is the middle position of arterial width, and *σ *is the standard deviation. The standard deviation, *σ*, is directly proportional to the arterial width. Ref [14] used the vascular outer diameter as measured by ultrasound to calculate Width_Index of the ADCW distribution which value was 0.355 ± 0.085. Therefore, in this study, the mean of the Width_Index (0.355) and *σ *were used to estimate the radial artery width and middle position, *X*_0 _in the envelope of the ADCW. According the estimated radial arterial width and *X*_0_, the left and right boundaries of the estimated radial arterial width were defined. The pressure waveform of the middle position was used as the reference pattern. Due to the resolution of the two-axis mechanism been 0.25 mm, we chose the segment that would be closest to the boundary.

**Figure 2:**
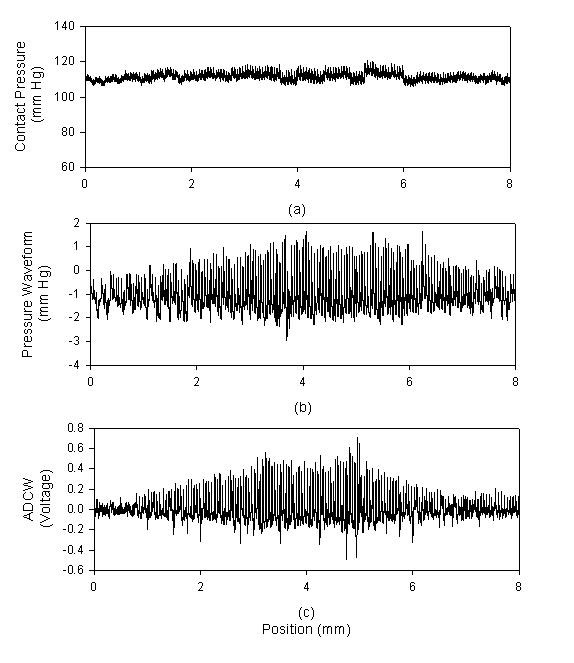
**In *X*-axis scanning, each movement was 0.25 mm, and each capture comprised 2000 points**. (a) The contact pressure, (b) The pressure waveform, (c) The arterial diameter changed waveform.

**Figure 3:**
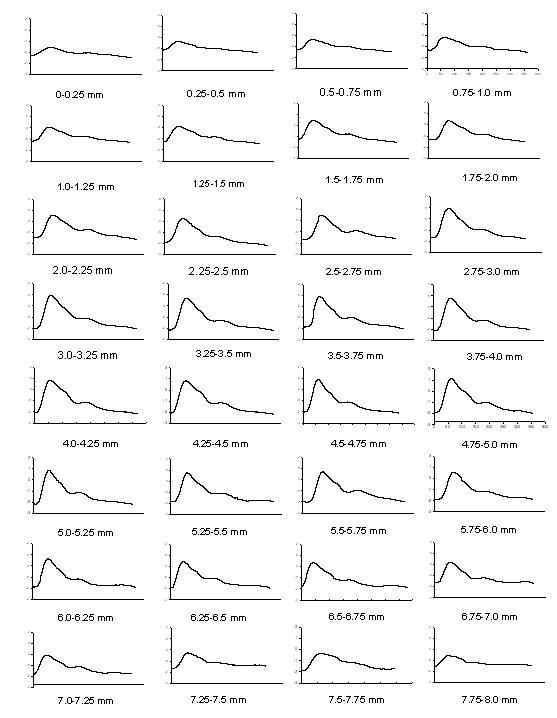
**The pressure waveforms are extracted from Fig. 2(b) at each segment**.

**Figure 4:**
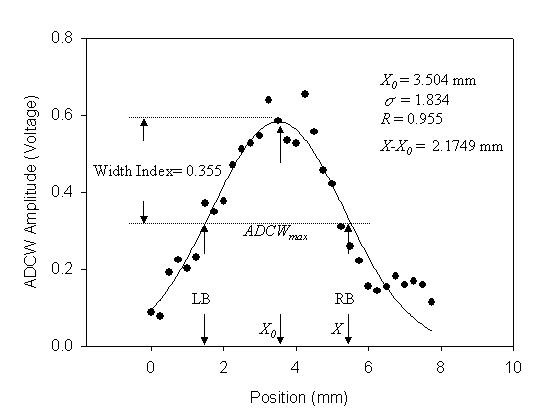
**Static ADCW-position relationship from the ADCW shown in Fig. 2(c)**. The dots and the line are the measured data and the regression function. LB and RB are the left and right boundaries of the estimated arterial width.

**Figure 5:**
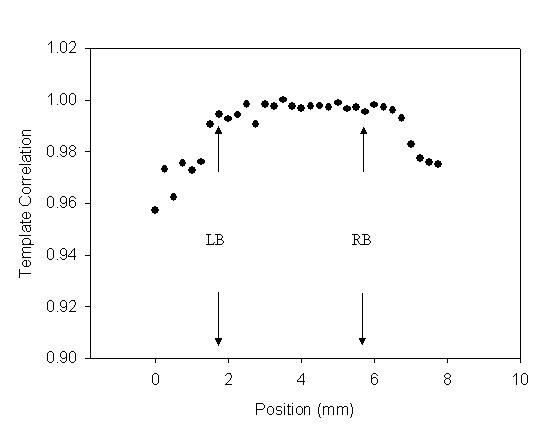
**The relationship between the sensor's position and the template correlation coefficients for Fig. 2(b) data**.

The recording signals of a subject were used to explain how to analyze the distortion of the pressure waveform affected by the sensor's location. In *X*-axis scanning, the measured contact pressure, pressure waveform and ADCW of a patient (sex: woman, age: 41 years, SBP: 94 mm Hg, DBP: 48 mm Hg, mean arterial pressure: 63 mm Hg, heart rate: 63 BPM) were shown in Fig. 2. In each step, a complete and perfect pressure waveform was shown in Fig 3. For the data shown in Fig. 2, we extracted the ADCW value from the averaged ADCW waveform peak-to-peak value at each moving step, as shown in Fig 4. We applied curve-fitting techniques to approximate the envelope of the ADCW to a Gaussian curve. The curve-fitting correlation coefficient, *R*, was 0.955. When the Width_Index was 0.355 and *σ *was 1.834, the estimated radial arterial width was 4.34 mm and *X_0 _*was 3.50 mm. In this case, because the middle position of the arterial width was located 3.5 mm, we used the pressure waveform locating at 3.5-3.75 mm segment as the reference pattern to compare over the pressure waveforms with Eq. (1) and (2). In each segment, the relationship between the sensor's position and the template correlation coefficients, *r*, was shown in Fig. 5. The segments 1.5-1.75 mm and 5.5-5.75 mm were defined as the left and right boundaries which template correlation coefficients were 0.990 and 0.997, respectively. The statistic of the template correlation coefficients within the arterial width is 0.996 ± 0.003.

### 2.3 Pressure Analysis in *Z*-axis

**Figure 6:**
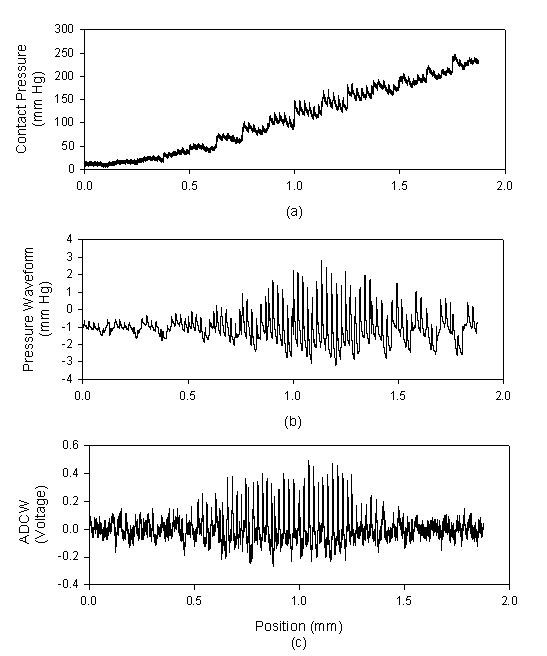
**In *Z*-axis scanning, each movement was 0.125 mm, and each capture comprised 2000 points**. (a) The contact pressure, (b) The pressure waveform, (c) The arterial diameter changed waveform.

**Figure 7:**
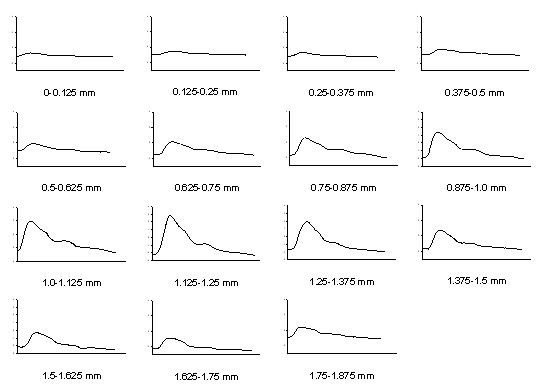
**The pressure waveforms are extracted from Fig. 6(b) at each segment**.

**Figure 8:**
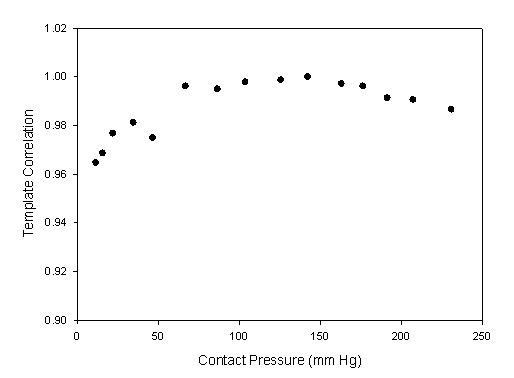
**The relationship between the sensor's contact pressure and the template correlation coefficients for Fig. 6(b) data**.

In the *Z*-axis, each step of the stepping motor was only 0.125 mm, and the recording time was 4 seconds. There were three to four complete cycles for the beat pulses. The pressure waveform with the maximum amplitude was used as the reference pattern. Figure 6 shows the measured contact pressure, pressure waveform and ADCW in the *Z*-axis scanning from the same patient in Fig 2. In each step, a complete and perfect pressure waveform was shown in Fig 7. We found that the maximum amplitude of the pressure waveform happened at the 1.125-1.25 mm position. Therefore, we used the pressure waveform locating at the 1.125-1.25 mm position as the reference pattern to compare over the pressure waveforms with Eq. (1) and (2). Fig 8 shows the relationship between the contact pressure and the template correlation coefficients, *r*, in each segment.

## 3. Results

**Figure 9:**
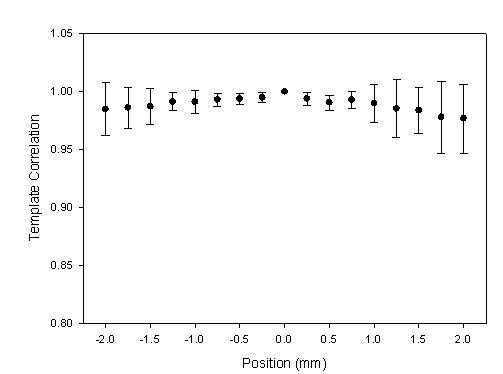
**The statistic data were the relation between the positions and the template correlation coefficients with the mean (solid circle) and standard deviation (bar) in *X*-axis scanning**. The segment of the reference pattern was set as the base position, 0 mm.

**Figure 10:**
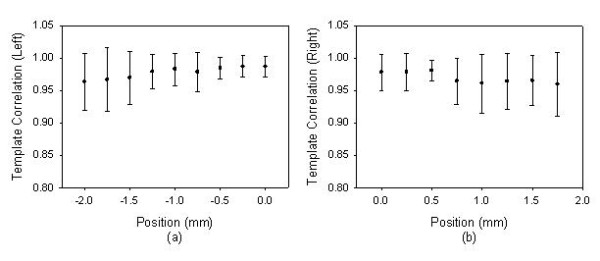
**When the sensor left the vascular boundary, the statistic data were the relation between the positions and the template correlation coefficients with the mean (solid circle) and standard deviation (bar)**. The left and right boundaries were set as the base position, 0 mm, which template correlation coefficients are 0.987 ± 0.016 and 0.978 ± 0.028, respectively. (a) The left side of radial artery, (b) The right side of radial artery.

In the *X*-axis scan, the distribution of the ADCW was used to estimate the radial arterial width and *X*_0 _according to the vascular geometry analysis [14]. The curve-fitting correlation coefficient, *R*, was 0.917 ± 0.079 (mean ± SD). The estimated radial arterial width was 3.64 ± 0.830 mm. The relations between the different distance of the middle position and the template correlation coefficients within 4 mm range are shown in Fig. 9 and Table 2 with the mean (solid circle) and standard deviation (bar). 0 mm represents the position of the reference pattern. Because the sensor was moved from left to right, a negative position represents the previous segment before the segment of the reference pattern. In order to understand the distortion degree of the pressure waveform when the sensor left the vascular boundary, we defined the left and right boundaries as the base position, 0 mm, which template correlation coefficients are 0.987 ± 0.016 and 0.978 ± 0.028, respectively. Figure 10 shows the statistic data with the mean (solid circle) and standard deviation (bar). The results for the left side of radial artery are shown in Fig 10(a), and the results for the right side of radial artery are shown in Fig 10(b). Because the sensor was moved from left to right, the prior position for the left boundary was set negative.

**Table 2:** The template correlation coefficients of each position in *X*-axis scan.

Position (mm)	-2.00	-1.75	-1.50	-1.25	-1.00	-0.75	-0.50	-0.25	0.0
*n*	42	42	42	42	42	42	42	42	42
Mean	0.985	0.986	0.987	0.991	0.991	0.993	0.994	0.995	1.00
± SD	0.028	0.018	0.016	0.008	0.010	0.006	0.005	0.004	0.00

Position (mm)	0.25	0.50	0.75	1.00	1.25	1.50	1.75	2.00	

*n*	42	42	42	42	42	42	42	42	
Mean	0.994	0.991	0.993	0.990	0.985	0.984	0.978	0.977	
± SD	0.005	0.006	0.008	0.016	0.025	0.020	0.031	0.030	

**Figure 11:**
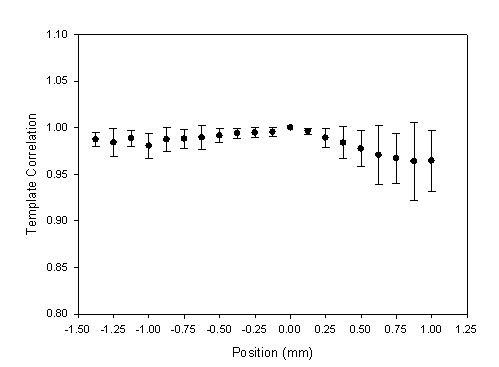
**The statistic data were the relation between the positions and the template correlation coefficients with the mean (solid circle) and standard deviation (bar) in *Z*-axis scanning**. The segment of the reference pattern was set as the base position, 0 mm.

**Figure 12:**
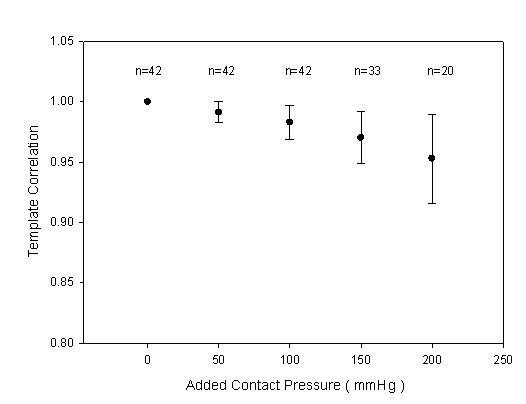
**The statistic data were the relation between excessive contact pressure and template correlation coefficients with the mean (solid circle) and standard deviation (bar)**. The contact pressure of the reference pattern was set as the base, 0 mm Hg, *n *was the sample number.

In the *Z*-axis scan, the contact pressure for the maximum amplitude was 161 ± 69 mmHg. The segment of the reference pattern was set as the base position, 0 mm, to understand the relation between the positions and the template correlation coefficients. These statistic data are shown in Fig. 11 and Table 3 with the mean (solid circle) and standard deviation (bar). Moreover, the number of the movement steps and the position of reference pattern were different for each subject. The sample numbers close to the beginning and ending positions would be less for some subjects. In Fig. 12, we also show the relation between excessive contact pressure and template correlation to describe that excessive contact pressure may produce alterations in arterial geometry. The contact pressure of the reference pattern was set as the base, 0 mm Hg. When the contact pressure was increased 50 mm Hg, the closest segment was selected from the Fig. 11. The template correlation coefficients were 0.991 ± 0.009, 0.983 ± 0.014, 0.970 ± 0.022, and 0.953 ± 0.037 at 50 mm Hg, 100 mmHg, 150 mmHg, and 200 mmHg, respectively.

**Table 3:** The template correlation coefficients of each position in *Z*-axis scan.

Position (mm)	-1.375	-1.25	-1.125	-1.00	-0.875	-0.75	-0.625	-0.50	-0.375	-0.25
*n*	17	21	27	38	42	42	42	42	42	42
Mean	0.987	0.984	0.989	0.980	0.987	0.988	0.989	0.991	0.994	0.995
± SD	0.008	0.015	0.008	0.013	0.013	0.010	0.013	0.007	0.005	0.005

Position (mm)	-0.125	0.0	0.125	0.25	0.375	0.5	0.625	0.75	0.875	1.00

*n*	42	42	42	42	42	39	34	26	21	13
Mean	0.995	1.00	0.996	0.989	0.984	0.978	0.971	0.967	0.964	0.964
± SD	0.005	0.00	0.003	0.010	0.017	0.019	0.031	0.027	0.042	0.032

## 4. Discussion

We had designed a two-axis mechanism and the modified sensor to detect the optimal measuring position by using vascular geometry analysis and arterial model for the pressure waveform measurement [14]. In the *X*-axis scan, the envelope of ADCW could be used to estimate the radial width with the Gaussian function. The *X*_0 _of the Gaussian function represented the middle position of the arterial width. The pressure waveform locating at *X*_0 _segment was used as the reference pattern. In Fig 3, we could find that this reference pattern may not have the maximum amplitude.

In the previous study, the vascular outer diameter, as measured by ultrasound, was used to calculate the Width_Index of the ADCW distribution. This yielded a value of 0.355 ± 0.085, with the mean value being used to estimate the radial arterial width. The measurement error, corresponding to the difference between the outer diameter and the estimated width, was 0.36 ± 0.23 mm [14]. Therefore, in this study, we also used the mean of the Width_Index (0.355) to roughly estimate the radial arterial width (3.64 ± 0.830 mm). For Fig. 2 subject, we used the Width_Index value and her *σ *value to estimate the width of radial artery. The template correlation coefficients between the left and right boundaries are approximate and above 0.99 in Fig. 5. We could find that the detected pressure waveforms were all vary similar when the sensor was placed within the radial arterial width. But, for all subjects, the estimated arterial widths are from 2.8 mm to 4.5 mm. The template correlation coefficients above 0.990 ± 0.016 are only within -1 mm and 1 mm positions in Table 2. Therefore, although, the optimal detective position has to be close to the middle of the radial arterial, the pressure waveform also has a good completeness with a template correlation coefficient of above 0.99 when the position was within ± 1 mm range. Moreover, the more leaving the middle position, the larger standard deviation. The results also represent the measuring stability being not enough. In Fig. 10, the template correlation coefficients of left and right boundaries are 0.987 ± 0.016 and 0.978 ± 0.028. When the sensor was moved far from the left or right boundaries, the template correlation coefficient decreased to about 0.95. We must emphasize that the Windth_Index was used to estimate the radial arterial width which was a rough value. Therefore, the statistic analyses of all subjects in the template correlation coefficients of left and right boundaries were different to the results of Fig. 5.

The radial artery is always close to the outside of the wrist. The palm points upwards and hand grasps the handgrip to allow the radial artery to appear easily and the radial styloid process is below the wrist's horizontal plane. In the *X*-axis scan, although the best contact pressure was achieved when it is held at a stable level, it always had some difference at the beginning and ending segments, as shown in Fig 2. Moreover, the right boundary of the radial artery is closest to the radial styloid process. When the sensor was moved downwards to apply the force at the right segments, the radial artery easily produced alterations in the arterial geometry through the soft silicone rubber of the sensor. Therefore, when the sensor moved from left to right at right hand, the template correlation coefficient of the left boundary was 0.987 ± 0.016. This was better than the right boundary correlation coefficient, which was 0.978 ± 0.028.

According to the arterial model, when the contact pressure was equal to the mean arterial pressure, there was an optimal matching between the sensor and artery [18,19]. The pressure waveform will have the maximum amplitude in this condition. However, in our study, the mean of the contact pressure of maximum amplitude was 161 mm Hg and it was larger than the whole mean arterial pressure (103 mm Hg). This is due to the contact area between the sensor and skin being very small, and is also probably attributable to the radial artery being loaded unilaterally by the sensor until the vessel moved downwards sufficiently for it to be supported by the radius. This explained why the contact pressure became larger than the mean arterial pressure.

In the Z-axis scan, when the sensor began just touching the artery, the pressure waveform was registered. When the amplitude of the measured pressure waveform changed from small to large and then back to small again, the measuring procedure would be ended. Figure 8 shows the template correlation coefficients in each moving step. The worse pressure waveforms happened in the beginning segments. The study's results also showed the contact pressure for the maximum amplitude being 161 ± 69 mmHg. Thus, the contact pressure of the maximum amplitude had a wide range. This meaning represented that the number of the movement steps and the position of reference pattern were different for each subject. Table 3 is the total statistic results which only show -1.375 mm to 1 mm range. But, the full samples, *n *= 42, were presented from -0.875 mm to 0.375 mm. Thus, the statistic analyses of all subjects in the template correlation coefficients of contact pressures were different to the results of Fig. 8.

The excessive contact pressure may produce alterations in arterial geometry and distort the arterial pressure waveform. In some conditions, although the arterial vessel had been compressed by the sensor, the detected pressure waveform didn't have too much distortion. Therefore, in Fig. 11, the mean of the template correlation coefficient decreases and the standard deviation becomes large when the sensor passes through the base position. Figure 12 focused on the results of the excessive contact pressure. The contact pressure with the maximum amplitude was considered as the criteria level. We compared the distortion degree of the pressure waveform over the criteria contact pressure. This phenomenon becomes very clear. Oppositely, in the segments of the lower contact pressure, the mean of the template correlation coefficient also decreases. But the standard deviation doesn't become large. Moreover, comparing the lower contact pressure, before the base segment, the template correlation coefficients (*r *= 0.988 ± 0.004) were better than those (*r *= 0.976 ± 0.012) in the higher contact pressure, after the base segment. These results also had been proven by Driscoll *et. al*. [12].

## 5. Conclusions

In this study, our contributions were that a two-axis mechanism and a modified sensor were used to quantitatively analyze the pressure waveform for locating the position and applying the force between the sensor and the radial artery. Although, the optimal detective position has to be close to the middle of the radial arterial, the pressure waveform also has a good completeness with template correlation coefficients of above 0.99 when the position was within ± 1 mm of the middle of the radial arterial range. When applying force, using the maximum amplitude as the criteria level, the template correlation coefficients were 0.991 ± 0.009, 0.983 ± 0.014, 0.970 ± 0.022, and 0.953 ± 0.037 at excessive 50 mm Hg, 100 mmHg, 150 mmHg, and 200 mmHg, respectively. We could find that the more excessive contact pressure, the worse pressure waveform.

## Appendix

### A. Flowchart of Measurement Procedure

**Figure 13:**
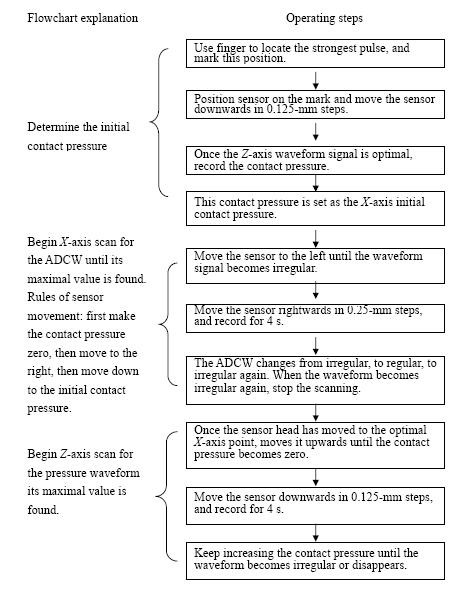
**Flowchart of our standard measurement procedure**.

Figure 13 shows the flowchart of measurement procedure.

### B. Vascular Geometry Analysis

**Figure 14:**
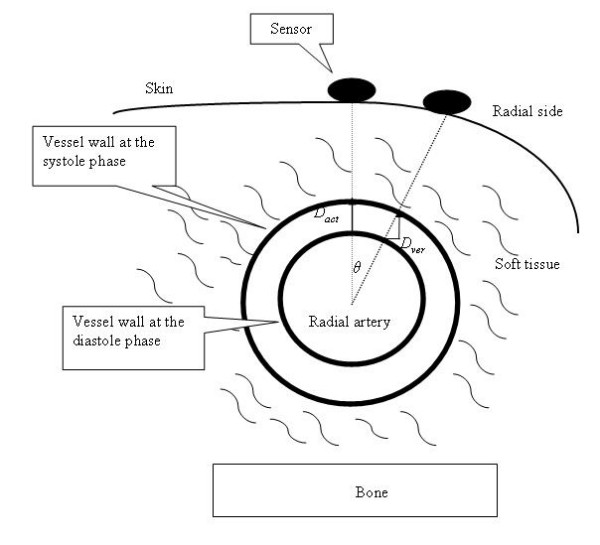
**Actual (*D*_*act*_) and vertical (*D*_*ver*_) displacements of the radial artery as detected by a sensor at different positions, where *D*_*ver *_= *D*_*act *_× *cos θ***.

The sensor can measure the ADCW as it moves across the radial artery. If the sensor is displaced from the middle of arterial width, the vertical component of the change in the arterial diameter is a triangular function, as shown in Fig. 14. When the artery expands, the actual displacement (*D*_*act*_) of the arterial wall is almost the same around its circumference. However, when the sensor is not positioned vertically above the middle of the artery, the relationship between the vertical displacement, *D*_*ver*_, and the actual displacement is given by *D*_*ver *_= *D*_*act *_× *cos θ*, where *θ *is the included angle between the middle position and the offset position. *D*_*ver *_decreases as this angle increases, and hence a strain gauge was used to measure changes in *D*_*ver *_. Therefore, as the sensor is scanned across the radial artery, *D*_*ver *_increases to *D*_*act *_and then decreases, with the measured ADCW amplitude in the same way. Therefore, the sensor was at the middle position of *X*-axis when the strain gauge detected the maximum ADCW.

## Competing interests

The authors declare that they have no competing interests.

## Authors' contributions

SHL participated in the design of the study, carried out the data process and statistical analysis analysis, drafted and revised the manuscript. CCT participated in acquisition of the data, and helped to revise the manuscript. All authors read and approved the final manuscript.
